# Stunting diagnostic and awareness: impact assessment study of sociodemographic factors of stunting among school-going children of Pakistan

**DOI:** 10.1186/s12887-020-02139-0

**Published:** 2020-05-19

**Authors:** Mahvish Ponum, Saadia Khan, Osman Hasan, Muhammad Tahir Mahmood, Asad Abbas, Mehwish Iftikhar, Reema Arshad

**Affiliations:** 1 School of Electrical Engineering and Computer Science, National University of Sciences and Technology, H/12 sector, Islamabad, Pakistan; 2 Department of Pediatrics, The Children’s Hospital & Institute of Child Health Multan, Multan, Pakistan; 3 Department of Computer Science, University of Engineering and Technology, Taxila, Pakistan; 4 Institute of Food Sciences and Nutrition, Bahauddin Zakariya University, Multan, Pakistan; 5 Department of Endocrinology and Metabolism, Services Hospital, Lahore, Pakistan

**Keywords:** Stunting, Stunting prevention, Stunting awareness, Stunting education

## Abstract

**Background:**

Stunting is a major public health issue in most of developing countries. Although, its worldwide prevalence is decreasing slowly but the number of stunted children is still rising in Pakistan. Stunting is highly associated with several long-term consequences, including higher rate of mortality and morbidity, deficient cognitive growth, school performance, learning capacity, work capacity and work productivity. To prevent stunting, we proposed Stunting Diagnostic and Education app. This app includes detailed knowledge of stunting and it’s all forms, symptoms, causes, video tutorials and guidelines by the Pediatricians and Nutritionists.

**Methods:**

A cross-sectional study has been conducted in schools of Multan District, Pakistan for the period of January 2019 to June 2019. Sample data of 1420 children, aged 4 to 18 years using three age groups, were analyzed by using SPSS version 21.0 to assess the prevalence of stunting and to analyze the risk factors associated with it in children under and over 5 age. Chi square test was applied in comparison with rural and urban participants and *p*-value < 0.05 was considered as significant. This study includes distribution of sociodemographic characteristics, parental education, working status of mothers, dietary patterns of school going children and prevalence of stunting in school going children. After getting study results, Stunting Diagnostic and Education app was developed according to the instructions of child experts and nutritionists.

**Results:**

354 (24.93%) participants were stunted out of 1420, 11.9% children were obese and 63.17% children were normal. Out of 354 stunted children, higher ratio of stunting was found in the age group of 8–11 years children with 51.98 percentage. 37.85% stunted children were found in the age group of 4–7 years and 10.17% stunting was found in the age group of 12–18 years children. It was observed in the study that male children were highly stunted than female with 57.91 and 42.09% respectively. Children living in rural areas were more stunted affected as compared to the children living in urban society with percentage 58.76 and 41.24 respectively.

**Conclusions:**

Our study concluded that 24.93% children were stunted, out of which, age group of 8–11 years children were highly stunted. The study showed that the literacy of mother or caregiver had high impact on children’s health. Therefore, Stunting Diagnostic and Education app was developed to educate mothers to diagnose stunting and to teach about the prevention of stunting.

## Background

Children survive and grow lively, develop and learn fast, play and get involved in activities by taking good nutrition while poor nutrition ruins children cognition and destroys their all working abilities. Stunting is ruinous result of poor nutrition in early childhood of children. Stunting affected children may gain impaired growth and development, may experience poor cognition and spread of repeated infection [1].

A child is defined as stunted if his height-for-age is below − 2 standard deviations (SD) from the median of World Health Organization (WHO) Child Growth Standards [2]. According to the statistics of WHO, globally, 149 million children suffered from stunting in 2018, 55% of stunted children reside in Asia [3] and its spread is higher than wasting and childhood overweight. In 2000, global stunting was recorded with 32.6 percentage in children under 5 age. Its rate has been declining slowly but steadily, the rate of stunting is declined to 21.9% in 2018, according to the data of UNICEF, WHO and World Bank Groups [4–6].

In Pakistan, the prevalence of stunting in children under five age is very high. Stunting under 5 age was 48% in 1965, it was declined to 36.3% in 1994 [7, 8]. In 2001, stunting was increased to 41.6 and 43.7% in 2011 [9]. According to the report of national nutrition survey 2018, very low progress was achieved to reduce stunting rate to 40.2% in 2018 which is very high to stunting threshold (> = 30%) [8].

Caregivers often lack the correct healthcare information, improper dietary counselling, breastfeeding, infant feeding, complementary feeding, maternal nutrition and improper childhood disease knowledge [10]. To improve these practices, in 2011, United Nations International Children’s Emergency Fund (UNICEF) and World Health Organization (WHO) jointly launched Infant and Young Children Feeding (IYCF) [11]. The aim of IYCF was to improve children growth and development. In 2012, for the period of 5 years (2012–2017), WHO initiated Pakistan Integrated Nutrition Strategy (PINS) to promote children nutrition counselling and education at various healthcare centers, community-based programs and child health days and to improve nutritional status of lactating and pregnant women [10]. PINS step forwarded to increase the knowledge of child caregivers and child service providers through civic education. Later, it also provided health trainings to schoolteachers. Aligning with PINS initiative, United Nations (UN) provided the support to the surveillance of nutrition and helping acute malnourished children for the period of 2013 to 2017 [12]. Pakistan with the help of international institutions, signed the Sustainable Development Goals (SDGs) to achieve the targets “end hunger and ensure access by all people” and “end all forms of malnutrition” by 2030 [13].

While the effect of stunting is very high. All these programs and most of the studies showed the underlying risk factors associated with stunting in children under 5 age including preterm birth, poor maternal nutrition, improper child feeding practices, ethnicity, birth interval of more than 24 months, mother’s low education and less awareness to nutrition, father’s low education, low consumption of vitamin A and environmental factors including improper sanitation [14–16]. Most of these risk factors are highly related to poverty [7]. This study also presents the prevalence of stunting and its associated factors in children ages between 4 years to 18 years.

Furthermore, the study [17] explained the stunting statistics of Pakistan for the period of 1991–2013 and showed that care in pregnancy, household assets, maternal and paternal education, fertility and open defecation had high impact on improving height for age z-scores (i.e. stunting reduction).

According to the study [17], maternal education is distinctive factor that affects the stunting. To the best of our knowledge, very little attempt is made through programs to educate mothers about stunting, maternal and child nutrition, in Pakistan [18]. No mHealth educating tool is proposed till the date. However, in this research, we focused on maternal education, maternal and child nutrition awareness and offering mHealth stunting diagnostic and education tool as a solution to reduce the prevalence of stunting. Moreover, we proposed Stunting Diagnostic and Education mHealth app to educate mothers about the diagnostic of all forms of stunting and to guide them about proper nutrition in antenatal period. This app provides the easy diagnostic of stunting based on symptoms, stunting prevention, nutritional practices for infants and young children, and nutritional video guidelines by the nutritionists and child experts.

## Methods

### Questionnaire design

Questionnaire was designed by 3 Nutritionist to get the data about height, weight, age, BMI, anthropometric measurements, physical activities, demographic characteristics and diet to analyze the prevalence of stunting and effects of factors on stunting. After development, all questions in questionnaire were analyzed by 3 Pediatrician to validate the data asked in questionnaire.

### Data collection

The data of 1420 school going children were collected from private and public schools of Multan district of Pakistan. These schools revealed low and high socioeconomics localities. The children with age 4 to 18 years participated in this study. The participants under 4 age and over 18 age and absent students were also excluded from the study, as Pakistan is getting major stunting population of ages 4 to 18 [8] .

The data were collected with the support of 4 researchers and 2 Nutritionist of The Children’s Hospital (CH) & Institute of Child Health Multan (ICHM) in Pakistan. Researchers calculated heights, weights and asked ages of children. They made the list of participants and provided the list to nutritionists. Nutritionists calculated height for age, obtained the stunted children from the lists and noted mild, moderate and severe stunted children.

### App development

**Fig. 1:**
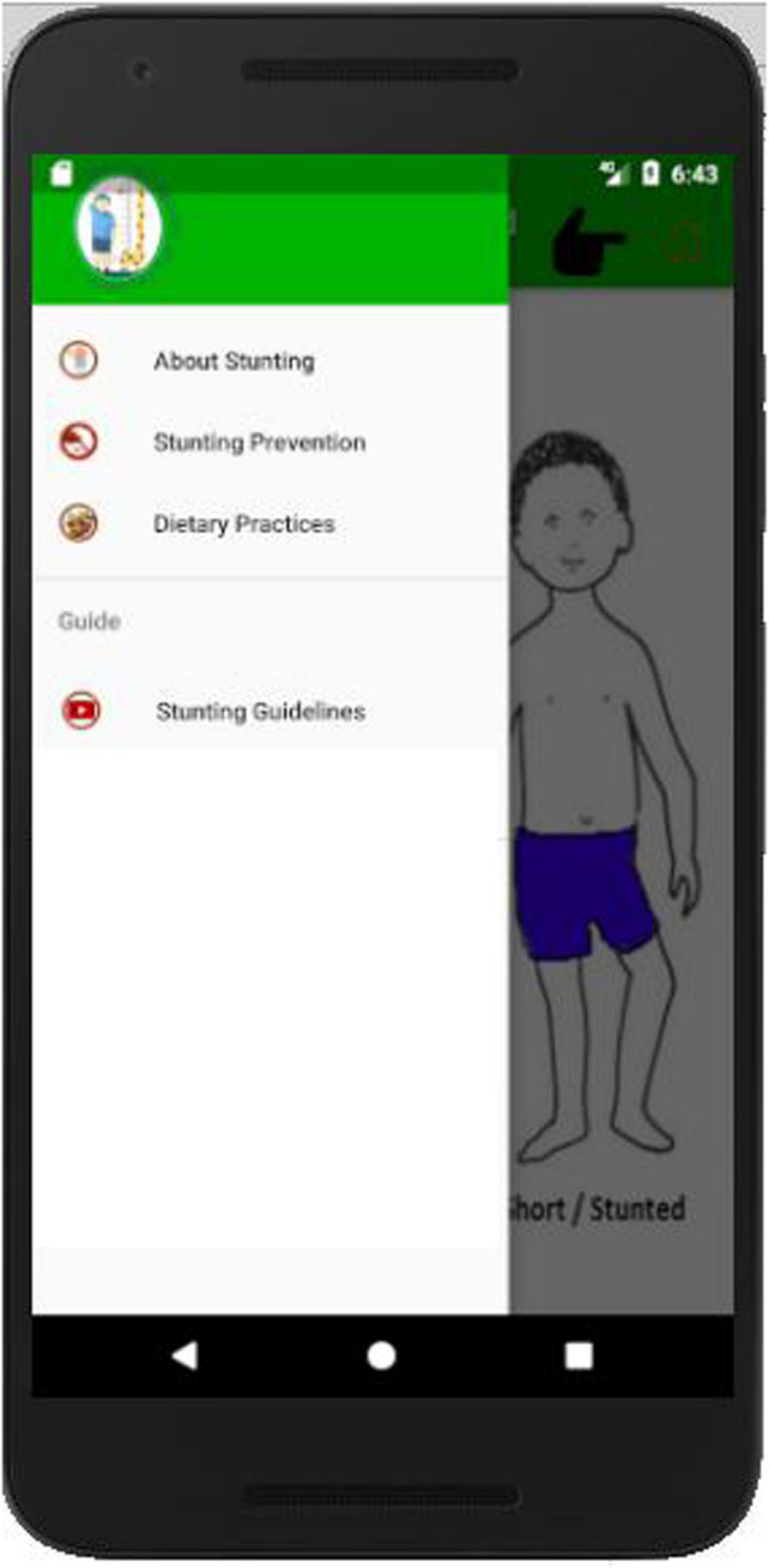
Modules of App are shown in figure to explain users about the detailed knowledge of stunting, its prevention, dietary practices and stunting guidelines. When a user tap on specific modules, it provides all necessary knowledge to user

The Stunting Diagnostic and Education app compasses 4 modules: stunting diagnostic, stunting prevention, dietary practices, stunting guidelines as shown in Fig. 1. The details of each module are explained in following subsections.

### Stunting diagnostic

**Fig. 2:**
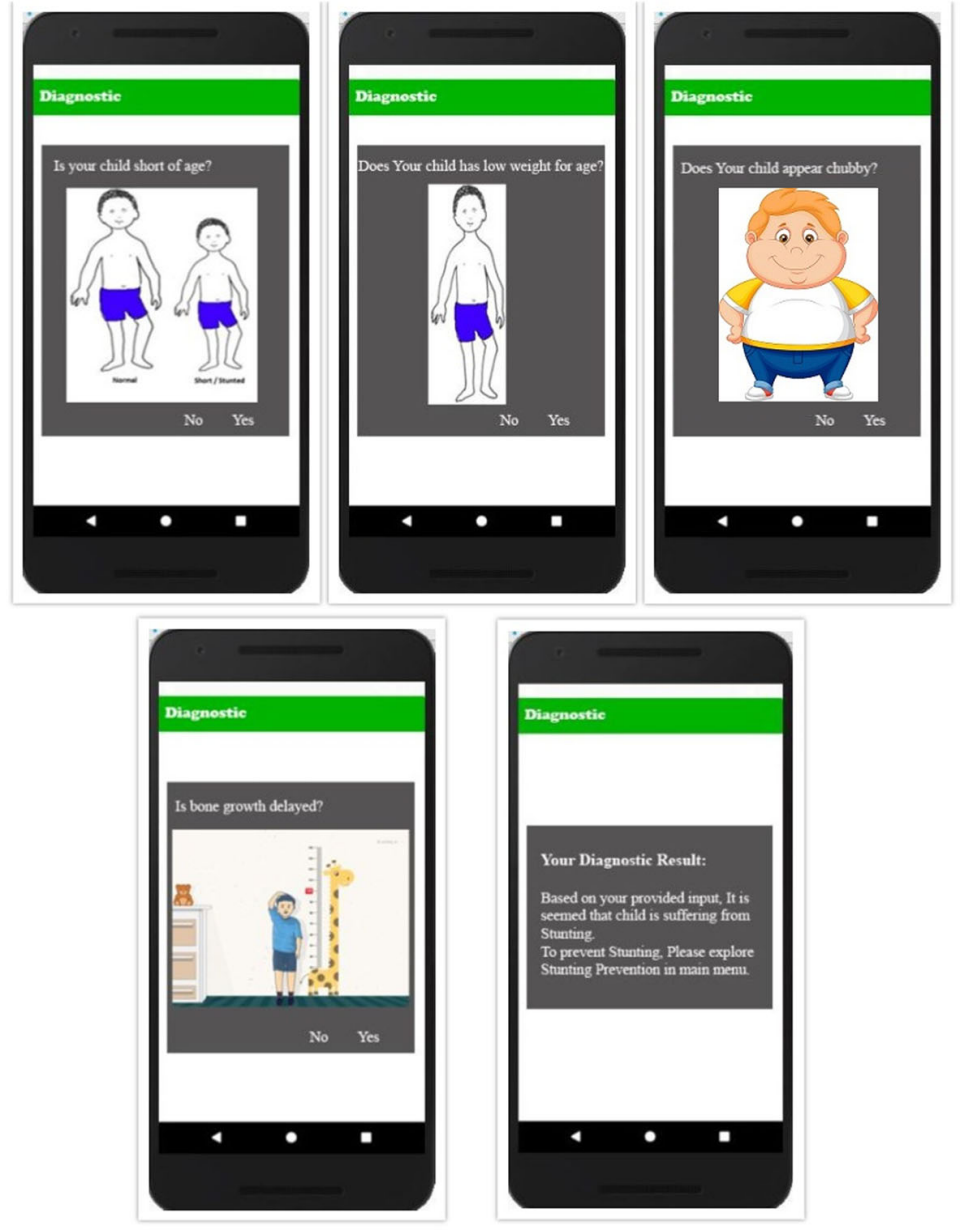
Diagnostic Test Interface shows the main interface of application. The diagnostic questions related to symptoms of stunting are asked from user to diagnose the stage of stunting

This module facilitates the caregiver to diagnose mild, moderate and severe stunting. The symptoms in the form of virtual patients are shown to caregivers for better understanding of signs of stunting. The module spots the stunting by querying simple questions from caregivers by showing them images of ill children. As, a mother starts the app, diagnostic appears promptly and provides easy navigation to caregiver. Interface itself guides the caregiver to navigate through the app. The diagnostic test interface is shown in Fig. 2.

### Stunting prevention

**Fig. 3:**
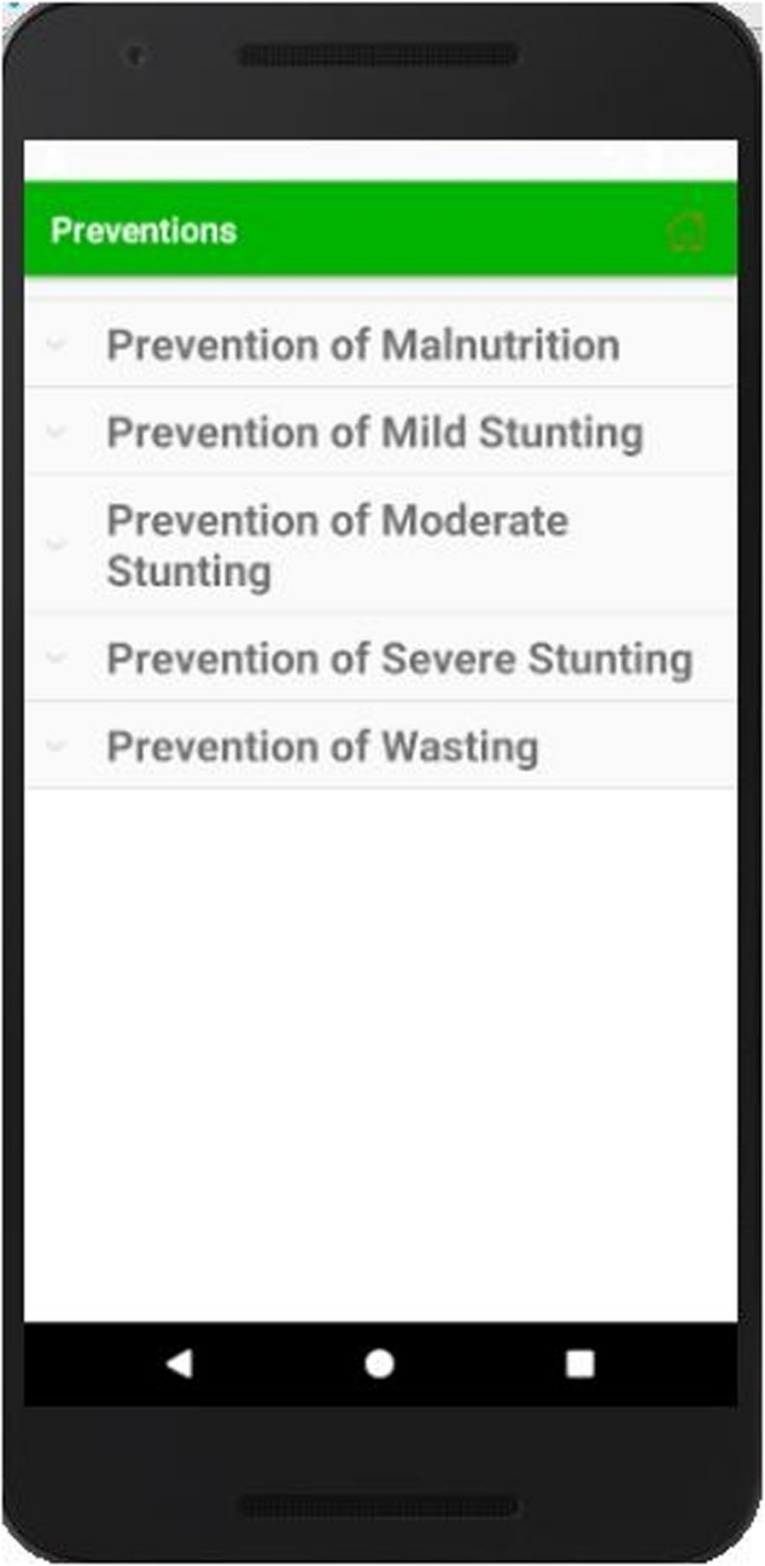
List of categories of disease is shown in this figure to provide the description of disease, symptoms of disease, causes of disease, preventive measures of disease and medical advice for the specific disease

**Fig. 4:**
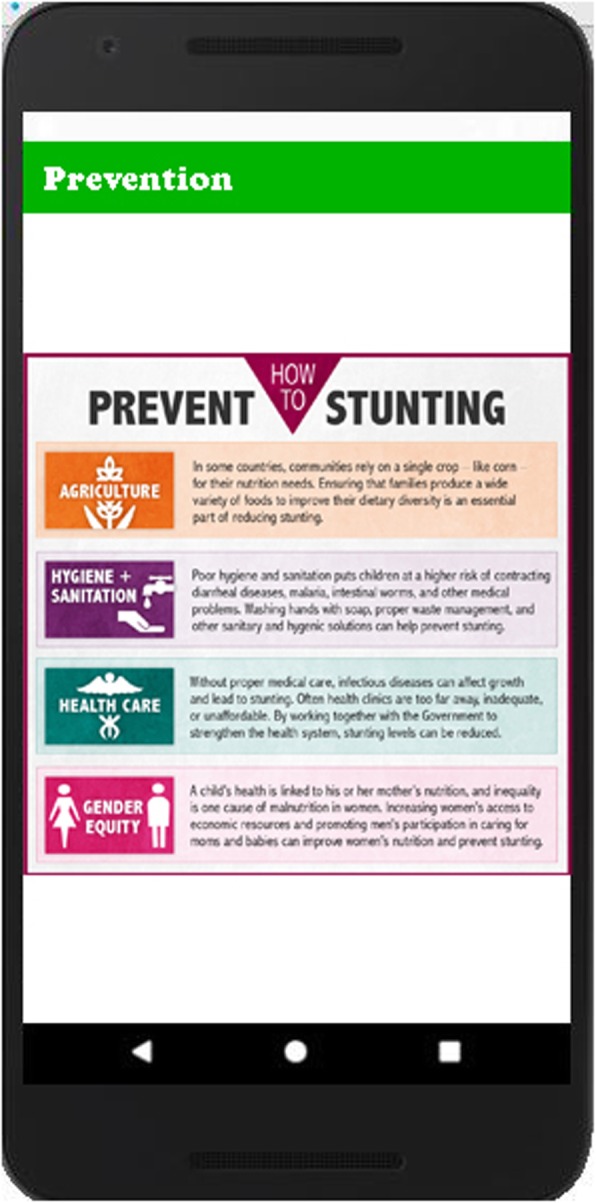
Prevention of Disease module provides the detailed knowledge to prevent the stunting. User learns the preventive measures to protect her kids from stunting

This module guides the caregivers to prevent mild, moderate and severe stunting. It focuses on prevention of infections through improved water, sanitation and hygiene, supplements of nutrient-rich foods and improving the quality of children’s diet to prevent stunting. Figure 3 shows the list of diseases to be prevented and their prevention in Fig. 4.

### Dietary practices

**Fig. 5:**
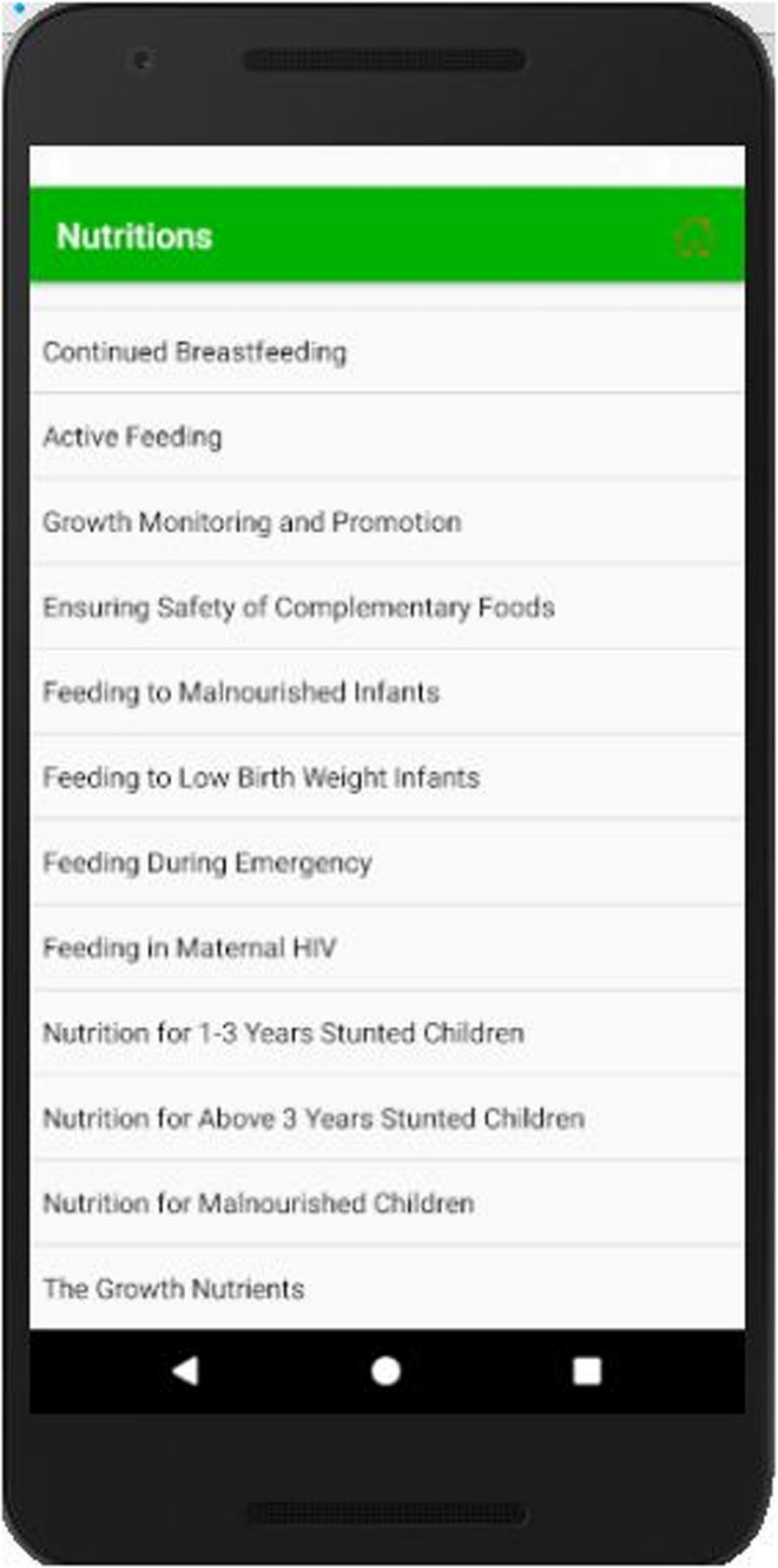
List of Nutrition shows the nutritional guidelines according to different age groups. It highlights the importance of breastfeeding, active feeding and provides essential knowledge about growth nutrients

**Fig. 6:**
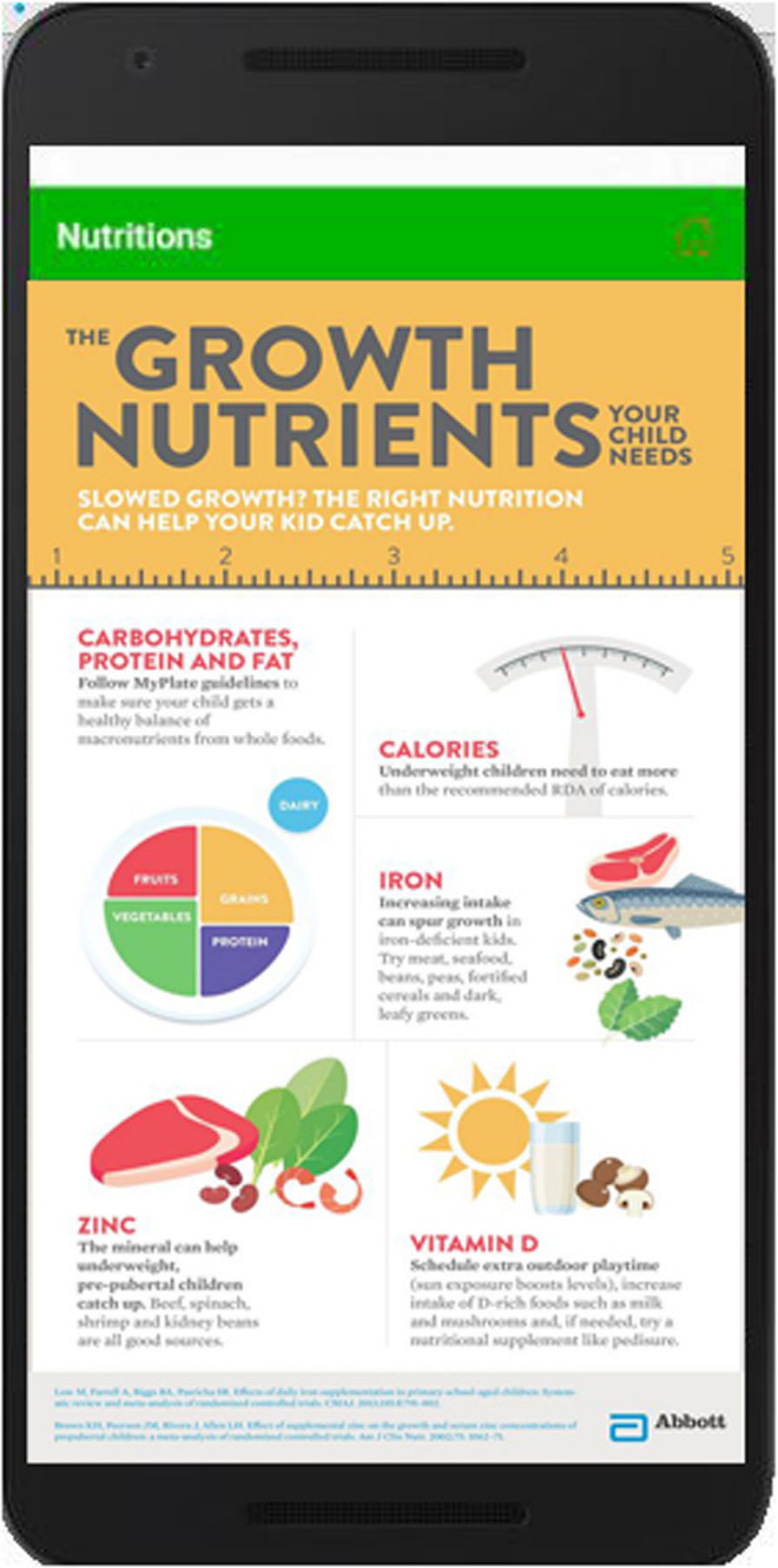
The Growth Nutrients are explained in this figure. When a user wants to learn about nutrition, he/she just taps on list menu-item and relevant menu is explained in detail

This module focuses on efficient diet during pregnancy, breastfeeding, continued breastfeeding, complementary feeding to infants and young children, consumption of vitamins and minerals (i.e. zinc, iron, calcium and vitamin A), usage of plant source foods (i.e. vegetables, fruits etc.) and consumption of animal source food (i.e. meat, eggs etc.) according to the ages of children. Figure 5 shows the list of nutrition and when user clicks “The Growth Nutrients”, app shows the details about the growth nutrients as shown in Fig. 6.

### Stunting guidelines

**Fig. 7:**
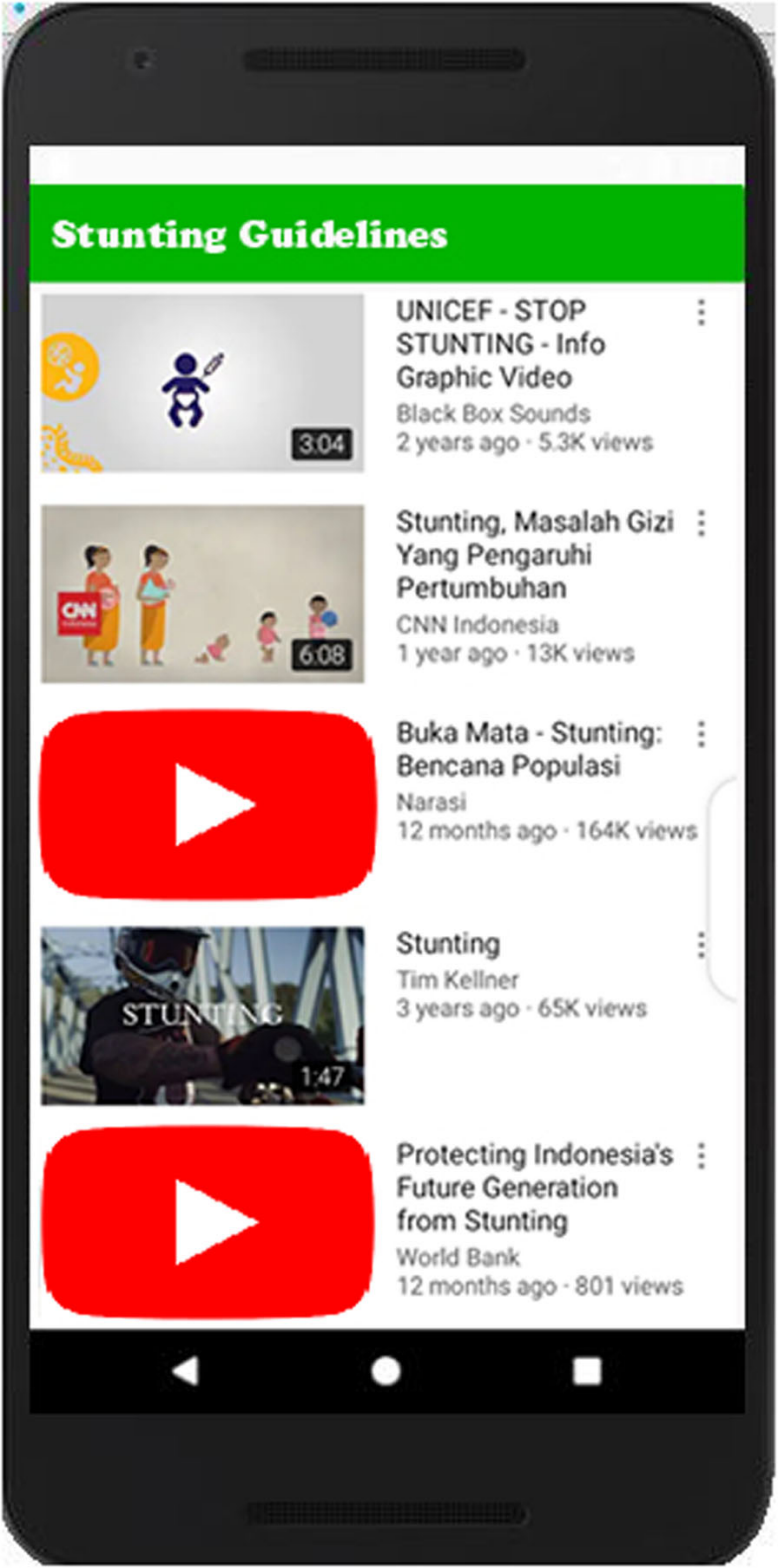
Stunting Guidelines module provides the detailed guidelines of stunting, its stages, its prevention and dietary guidelines by the child expert

This module consist of videos tutorials of stunted children to explain all question including what is stunting, what are causes of stunting, how to treat stunted children, how to look after a stunted child at home, what kind of nutrition, caregiver should provide to stunted child, which things should be avoided to give to stunted child and it provides the video guidelines on dos and don’ts of stunting. Figure 7 shows the stunting video guidelines.

## Results

Table 1 shows various sociodemographic factors of school going children, determined in questionnaire. Total 1420 children participated in survey. The percentage of male participant was 52.11 and female participants’ percentage was 48.89. The participants living in rural localities were more than participants living in urban areas with percentage 75.35 and 24.65 respectively.

**Table 1:** Distribution of Sociodemographic factors of school going Children is shown in table. It shows the impact of kids’ residence, their mothers’ qualification, father’s occupation, family size and number of siblings, on child’s health

Characteristics	Frequency	Percentage
**Gender**		
Male	740	52.11
Female	680	48.89
**Residence**		
Rural	1070	75.35
Urban	350	24.65
**Father’s Literacy**		
Literate	604	42.54
Illiterate	816	57.46
**Mother’s Literacy**		
Literate	551	38.80
Illiterate	869	61.20
**Mother working status**		
House wife	1207	85
Outside home working	213	15
**Father occupation**		
Laborer	567	39.93
Farmer	58	4.08
Govt. employer	452	31.83
Shopkeeper	90	6.34
Landlord	48	3.38
Others	205	14.44
**Family size**		
> 5	1046	73.66
< 5	374	20.34
**Number of siblings**		
No	157	11.06
1–3	583	41.06
> 3	680	47.88

Literacy rate of participants’ fathers and mothers was low and illiteracy rate was high. The literacy percentage of their fathers was 42.54 and mothers’ literacy percentage was 38.80. The illiteracy percentage of their fathers 57.46 and their mothers’ illiteracy percentage was 61.20. When the working status of father of each child was inquired, the study found that most of fathers’ occupation was labor with 39.93 percentage and 31.83% fathers were government employee. 73.66% participants belonged to families having family size > 5 and 47.88% of participants having > 3 number of siblings.

Prevalence of stunting is determined in Table 2. Among 1420 children, 63.17% children were normal, 24.93% children were stunted and 11.90% children were overweight and obese. The study shows that males were highly affected with stunted with 57.91 percentage and stunted female percentage was 42.09. 58.76% stunted children were living in rural areas and 41.24% stunted children were living in urban areas as depicted in Table 2.

**Table 2:** Prevalence of Stunting in school going children is shown in table to show the ratio of stunted children, obese and overweight children, stunted male and female ratio and ratio of children living in urban and rural areas

Characteristics	Frequency	Percentage
Normal	897	63.17
Overweight and obese	169	11.90
Stunted	354	24.93
Stunted Male (*N* = 354)	205	57.91
Stunted Female (*N* = 354)	149	42.09
Stunted children living in rural areas (*N* = 354)	208	58.76
Stunted children living in urban areas (*N* = 354)	146	41.24

Table 3 shows the distribution of stunting according to different age groups. It has been observed in study that participants of age group of 8–11 years were highly stunted with 51.98 percentage than 4–7 years children with 37.85 percentage and 12–18 years children with 10.17 percentage. Among 8–11 years age group of participants, 58.06% children were suffering from mild stunting, 52.57% children were facing moderate stunting and 44.19% were diagnosed with severe stunting.

**Table 3:** Distribution of Stunting is calculated according to different age groups. Ratio of children with mild, moderate and severe stunting is calculated according to 3 ages group including 4–7 years, 8–11 years and 12–18 years

Age groups	Total stunted participants *N* = 354	Mild Stunting (−2SD - < −1SD) *N* = 93	Moderate Stunting (−3SD - < −2SD) *N* = 175	Severe stunting (<−3SD) *N* = 86
4–7 years	134 (37.85%)	21 (22.58%)	73 (41.71%)	40 (46.51%)
8–11 years	184 (51.98%)	54 (58.06%)	92 (52.57%)	38 (44.19%)
12–18 years	36 (10.17%)	18 (19.35%)	10 (5.72%)	8 (9.3%)

The impact of sociodemographic factors was noticed carefully in the study. Most of children suffering from moderate and severe stunting were those whose fathers’ and mothers’ education was low. Father’s occupation was laborer and government employer and their mother’s work outside. Family size is also a major factor that effects the health of children. It was analyzed in the study that most of the children were severe stunted whose family size was greater than 5 with 73.66 percentage and their number of siblings was greater than 3 with 47.88 percentage.

Dietary patterns of each child were examined in the study. It was found that 55.36% stunted children usually skipped the breakfast and only 12.66% non-stunted children skipped the breakfast. The consumption of fruits, vegetables, eggs, meat, pulses and dairy items was very low in stunted children, as shown in Table 4. On the other hand, non-stunted children usually consume basic food group items.

**Table 4:** Dietary Patterns of non-stunted and stunted participants are analyzed to see the impact of breakfast, fruits, vegetables, meat, eggs, pulses and dairy products, on the health of children

Characteristics	Non-stunted (***n*** = 1066)		Stunted (***n*** = 354)		***P***-value
	No.	(%)	No.	(%)	
**Breakfast**					
Skip	135	12.66	196	55.36	0.04
Usually eat	931	87.34	158	44.64	> 0.05
**Basic food groups**					
Fruits	119	11.16	22	6.21	0.04
Vegetables	531	49.82	102	28.81	> 0.05
Egg	98	9.19	34	9.60	< 0.05
Meat	92	8.63	76	21.46	0.16
Pulses	106	9.95	84	23.72	< 0.05
Dairy	120	11.25	36	10.16	0.28

Table 5 shows the distribution of dietary patterns of stunted children in rural and urban areas. It shows that 66.35% stunted children skip breakfast in rural areas and in urban areas, 39.72% stunted children skip breakfast which is not good for health. Fewer stunted children usually eat breakfast in rural areas with 33.65 percentage and in urban areas, majority of stunted children usually eat breakfast with 60.28 percentage. The consumption of vegetables, eggs, meat, pulses and dairy products is higher in urban areas as compared to rural areas.

**Table 5:** Distribution of dietary patterns of stunted children is calculated with respect to Rural and Urban areas. The results are derived to analyze the outcomes of different diet on the health of children living in urban and rural areas

Characteristics	Rural (***n*** = 208)		Urban (***n*** = 146)		***P***-value
	No.	(%)	No.	(%)	
**Breakfast**					
Skip	138	66.35	58	39.72	0.05
Usually eat	70	33.65	88	60.28	> 0.05
**Basic food groups**					
Fruits	9	4.33	13	8.90	0.06
Vegetables	56	20.92	46	31.51	< 0.05
Egg	13	6.25	21	14.38	0.12
Meat	33	15.86	43	29.45	> 0.05
Pulses	45	21.63	39	26.71	0.05
Dairy	13	6.25	23	15.75	0.08

## Discussion

After getting the results of population, it is analyzed that which knowledge should be added in the app to educate mother about stunting. After analyzing the results, the stunting diagnostic, stunting prevention, dietary practices and stunting guidelines are added into the app. Furthermore, the evaluation of the usability of app is analyzed.

The usability study should have at least 10 participants [19], so, to evaluate the usability of app 15 mothers with stunted patients were gathered from Multan’s urban areas. The app was installed in all mothers’ cell phones. All mothers were asked to start the stunting diagnostic and education app. As they tapped the icon of app, the diagnostic test was appeared on the interface of cell phone and question related to stunting were asked to mothers. The mothers diagnosed mild and moderate stunting the most in their children using app. After diagnosis, app instructed to explore prevention of stunting and to explore nutrition for healthy diet.

When asked about the app’s usability, most of the mothers said that app provides easy navigation and it is very user-friendly. Most of the mothers liked its simplicity and ease of access of all features. Mothers said that they liked its diagnostic test feature, nutrition feature and video guidelines the most. They suggested to publish it over Google play store and to make its access free all over the Pakistan. They further suggested to spread it among all health agencies of Pakistan, so that each caregiver can get benefit from this app.

## Conclusions

The excision of children stunting is very challenging as it is affecting millions of children of Pakistan. To prevent stunting, various measures has been taken in Pakistan but the results were not satisfactory. The study concluded that stunting prevalence was high in males than females. Most of stunted children were living in rural areas. The literacy rate of stunted children’s mother and father was low. The large family size also affected the health of children. When dietary patterns of stunted children were analyzed then it is found that majority of stunted children skip their breakfast and the consumption of fruits, vegetables, eggs (basic food group) was very low. So, poor diet, poor sanitation practices and hygiene, poverty, poor maternal nutrition in pregnancy, not exclusively breastfeeding and repeated infections were other common causes found in stunted children. To prevent stunting, the Stunting Diagnostic and Education app was developed to teach mothers about healthy diet intake during pregnancy, supplementation of iron-folic acid during pregnancy to reduce the risk of stunting in children, complementary feeding in young children, water, household sanitation and hygiene practices, pulses, alternative source of protein, energy, iron and zinc, disadvantages of bottle feeding, misconceptions such as concepts of hot and cold food etc.

The app proved to be very useful and mothers liked its all features. This app would be made available to all mothers over Google play store, in future. This version of app supports English language, the next version of app would support Urdu language too, for Pakistani mothers with low education. This version neither include any prescription or any medical advice related to medicine dosage nor it is any alteration to medical processes. It is simply an education tool for mothers to better understand about stunting and its prevention.

## Data Availability

Data will be provided to each reader on demand. Reader can request via email.

## Abbreviations

U5: Under Five
UN: United Nations
WHO: World Health Organization
SDGs: Sustainable Development Goals
IYCF: Infant and Young Children Feeding
PINS: Pakistan Integrated Nutrition Strategy
UNICEF: United Nations International Children’s Emergency Fund

## References

[1] Nutrition: Stunting in a nutshell https://www.who.int/nutrition/healthygrowthproj_stunted_videos/en/.

[2] Vonaesch P, Tondeur L, Breurec S, Bata P, Nguyen LBL, Frank T (2017). Factors associated with stunting in healthy children aged 5 years and less living in Bangui (RCA). PLoS One.

[3] Children Malnutrition*,* World Health Organization https://www.who.int/gho/child-malnutrition/en/.

[4] Malnutrition rates remain alarming: stunting is declining too slowly while wasting still impacts the lives of far too many young children, April 2019 https://data.unicef.org/topic/nutrition/malnutrition/.

[5] Levels and trends in child malnutrition (2019). UNICEF/WHO/World Bank Group joint child malnutrition estimates, Key findings of the 2019 edition.

[6] Levels and trends in children malnutrition (2018). UNICEF/WHO/World Bank Group joint child malnutrition estimates, Key findings of the 2018 edition.

[7] Stunting in Pakistan (2017). A Trend Analysis of Underlying Factors by 2030, Inter-agency regional analysts network, ASIA.

[8] National Nutrition Survey. 2018, Key findings report, nutrition wing, ministry of national health services, Regulations and Coordination Government of Pakistan, vol. 2018.

[9] *Prevalence of stunting, height for age (% of children under 5)* https://data.worldbank.org/indicator/SH.STA.STNT.ZS.

[10] Pakistan Integrated Nutrition Strategy (PINS) (2011). Operational framework /plan.

[11] Programming Guide (2011). Infant and young child feeding, UNICEF.

[12] *Nutrition*, United Nations, 2017 https://www.un.org.pk/nutrition/.

[13] United Nations Development Programme, Goal 2: Zero Hunger. https://www.undp.org/content/undp/en/home/sustainable-development-goals/goal-2-zero-hunger.html.

[14] Danaei G, Andrews KG, Sudfeld CR, Fink G, McCoy DC, Peet E (2016). Risk factors for childhood stunting in 137 developing countries: a comparative risk assessment analysis at global, regional, and country levels. PLoS Med.

[15] Tariq J, Sajjad A, Zakar R, Zakar MZ, Fischer F (2018). Factors Associated with Undernutrition in Children under the Age of Two Years: Secondary Data Analysis Based on the Pakistan Demographic and Health Survey 2012–2013. Nutrients..

[16] Farid-ul-Hasnain S, Sophie R. Prevalence and risk factors for Stunting among children under 5 years: a community based study from Jhangara town, Dadu Sindh. J Pakistan Med Assoc. 2010;60(1). 20055279

[17] Headey D, Hoddinott J, Park S. Drivers of nutritional change in four South Asian countries: a dynamic observational analysis. Maternal Child Nutr. 2016;12(Suppl. 1). 10.1111/mcn.12274 PMC5084796 27187917

[18] Ponum M, Hasan O, Khan S (2019). EasyDetectDisease: An Android App for Early Symptom Detection and Prevention of Childhood Infectious Diseases. Interact J Med Res.

[19] Nielsen J, Landauer T (1993). “A mathematical model of the finding of usability problems” INTERACT '93 and CHI '93 conference on human factors in computing systems.

